# Supplementary material for: Control of magnetic states and spin interactions in bilayer CrCl3 with strain and electric fields: an ab initio study
Source: Sci Rep. 2023 Apr 1;13:5336. doi: 10.1038/s41598-023-32598-1 (PMC10067849; doi:10.1038/s41598-023-32598-1)
Supplement: Supplementary file 1 — Supplementary Information. [file 41598_2023_32598_MOESM1_ESM.pdf]

Supplementary Information for Control of magnetic states and spin interactions in bilayer $CrCl_3$ with strain and electric fields: An ab initio study

Ali Ebrahimian,[1,2] Anna Dyrdał,[1] and Alireza Qaiumzadeh[3]

[1]*Department of Mesoscopic Physics, ISQI, Faculty of Physics, Adam Mickiewicz University, ul. Uniwersytetu Poznanskiego 2, 61-614 Poznan, Poland*
[2]*School of Physics, Institute for Research in Fundamental Sciences (IPM),Tehran 19395-5531, Iran*
[3]*Center for Quantum Spintronics, Department of Physics, Norwegian University of Science and Technology, NO-7491 Trondheim, Norway*

I. ROBUSTNESS OF RESULTS AGAINST DIFFERENT U VALUES

Figure I.1 shows the robustness of the magnetic ground-state against different Hubbard U values. In fact, the sign of magnetic anisotropy (MAE $= E_{\rm IN} - E_{\rm OUT}$) and the magnetic exchange interaction ($E_{\rm AFM}$ - $E_{\rm FM}$) are not changed as we change the onsite Hubbard parameter.

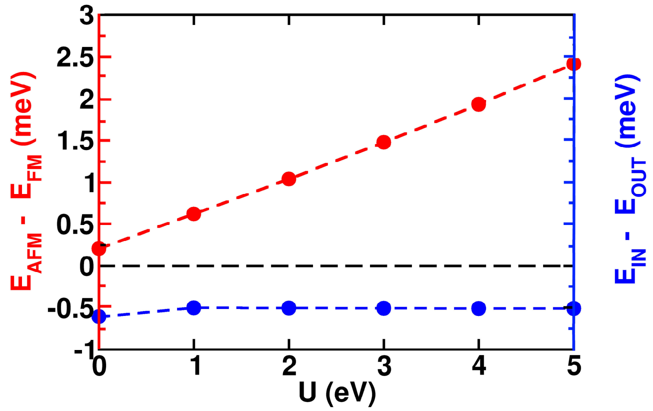

FIG. I.1. (Color online) Energy differences between AFM and FM states and MAE under different Hubbard U parameters.

II. STRAIN-INDUCED CHANGES IN THE BONDING ANGLE AND BONDING LENGTH

The strain fields can alter the bonding angle, $\alpha_{\mathrm{Cr-Cl-Cr}}$, and bonding length, $L_{\mathrm{Cr-Cl}}$. For all FM, A-type AFM, and G-type AFM configurations, the tensile strain increases $\alpha_{\mathrm{Cr-Cl-Cr}}$, while this angle decreases under compressive strain. Figure II.2 shows that the Cr-I bonding length slightly changes in the presence of strain fields.

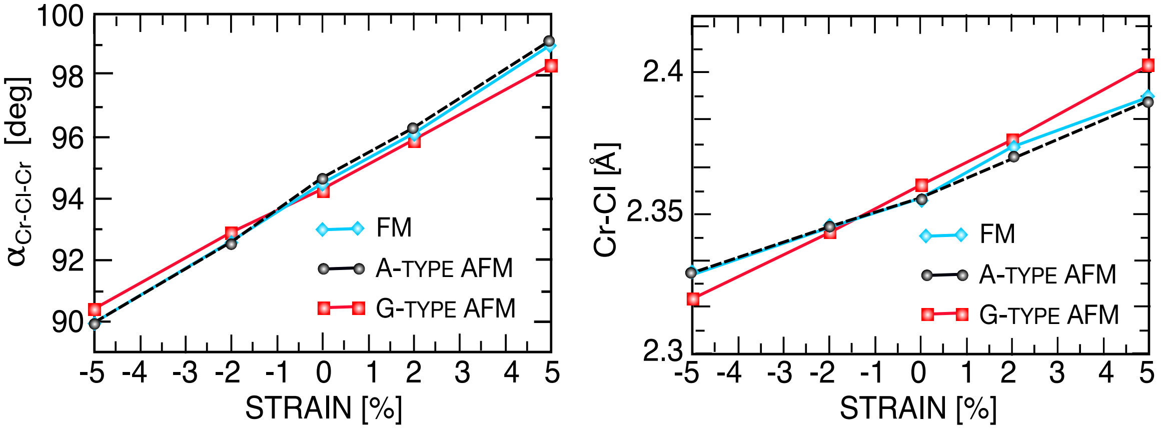

FIG. II.2. (Color online) The Cr-Cl-Cr bonding angle ($\alpha_{Cr-Cl-Cr}$) (left) and the Cr-Cl bonding length (L_{Cr-Cl}) (right) in bilayer $CrCl_3$ under compressive (negative sign) and tensile (positive sign) biaxial strains. The α angle decreases with increasing compressive strain, while it increases toward a straight angle with increasing the tensile strain.
